# Alcohol use and tuberculosis clinical presentation at the time of diagnosis in Puducherry and Tamil Nadu, India

**DOI:** 10.1371/journal.pone.0240595

**Published:** 2020-12-17

**Authors:** Carolyn K. Kan, Elizabeth J. Ragan, Sonali Sarkar, Selby Knudsen, Megan Forsyth, Muthaiah Muthuraj, Kumar Vinod, Helen E. Jenkins, C. Robert Horsburgh, Padmini Salgame, Gautam Roy, Jerrold J. Ellner, Karen R. Jacobson, Swaroop Sahu, Natasha S. Hochberg

**Affiliations:** 1 Section of Infectious Diseases, Boston University School of Medicine and Boston Medical Center, Boston, Massachusetts, United States of America; 2 Department of Preventive and Social Medicine, Jawaharlal Institute of Postgraduate Medical Education and Research, Gorimedu, Puducherry, India; 3 Intermediate Reference Laboratory, Government Hospital for Chest Diseases, Gorimedu, Puducherry, India; 4 Department of Pulmonary Medicine, Jawaharlal Institute of Postgraduate Medical Education and Research, Gorimedu, Puducherry, India; 5 Department of Biostatistics, Boston University School of Public Health, Boston, Massachusetts, United States of America; 6 Departments of Epidemiology and Global Health, Boston University School of Public Health, Boston, Massachusetts, United States of America; 7 Division of Infectious Diseases, Department of Medicine, New Jersey Medical School, Rutgers University, Newark, New Jersey, United States of America; Jamia Hamdard, INDIA

## Abstract

**Setting:**

Alcohol use increases the risk of tuberculosis (TB) disease and is associated with worse outcomes.

**Objective:**

To determine whether alcohol use affects TB severity at diagnosis in a high-burden setting.

**Design:**

Participants were smear-positive people living with TB (PLWTB) in India. Disease severity was assessed as 1) high versus low smear grade, 2) time to positivity (TTP) on liquid culture, 3) chest radiograph cavitation, and 4) percent lung affected. Alcohol use and being at-risk for alcohol use disorders (AUD) were assessed using the AUDIT-C. Univariable and multivariable analyses were conducted.

**Results:**

Of 1166 PLWTB, 691 (59.3%) were drinkers; of those, 518/691 (75.0%) were at-risk for AUD. Drinkers had more lung affected than non-drinkers (adjusted mean difference 10.8%, p<0.0001); this was not significant for those at-risk for AUD (adjusted mean difference 3.7%, p = 0.11). High smear grade (aOR 1.0, 95%CI: 0.7–1.4), cavitation (aOR 0.8, 95%CI 0.4–1.8), and TTP (mean difference 5.2 hours, p = 0.51) did not differ between drinkers and non-drinkers, nor between those at-risk and not at-risk for AUD.

**Conclusions:**

A large proportion of PLWTB were drinkers and were at-risk for AUD. Alcohol drinkers had more lung affected than non-drinkers. Studies are needed to explore mechanisms of this association.

## Introduction

Despite global efforts, tuberculosis (TB) remains a significant cause of death and long-term disability [1]. Clinical characteristics at the time of TB diagnosis are important because more advanced disease at presentation predicts worse clinical outcomes, and cavitation and higher smear grade are associated with increased infectiousness and transmission risk [2, 3]. Percent lung affected, when accounting for cavitation, has been found to predict delays in smear conversion among pulmonary TB patients [4]. Alcohol use has been associated with both progression to TB disease and poor TB treatment outcomes [5–8]. Studies have shown an association between alcohol use and both lung cavitation and smear positivity, but these studies are limited to low-burden settings and results have been conflicting [9–11].

Alcohol use has been associated with altered immune responses which may lead to higher disease burden and increased lung pathology. From a biologic perspective, alcohol consumption has been shown to impact both innate and adaptive immune responses critical to controlling *Mycobacterium tuberculosis* (Mtb) [12], including a decrease in macrophage [13, 14] and neutrophil function [15] and alteration in cytokine production [16]. Alcohol consumption in young mice infected with *Mtb* has been shown to lead to IFN-α production and reduced survival [17]. Multiple behavioral pathways of delays in seeking care may also potentially lead to increased lung damage [8, 18].

Alcohol use disorder (AUD) is an increasingly global issue, with total alcohol consumption positively associated with a country’s wealth [19]. India has transitioned from a traditionally “dry” country to a 57% increase in *per capita* alcohol consumption between 2005 and 2016, where alcohol consumption rose from 2.4 liters *per capita* to 5.7 liters *per capita*, accordingly [19]. In India, alcohol is consumed primarily by males, with the 2016 AUD prevalence at 9.1% in males compared to 0.5% in females [19]. A study conducted in Tamil Nadu on TB patients from ten tuberculosis units (TUs), a sub-district organizational structure for TB care, found a 44% alcohol use prevalence amongst males and no use reported by females, with 16% of all users reporting alcohol dependence on the Alcohol Use Disorders Identification Test (AUDIT) [20]. Another study of TB patients in Puducherry found that 59% of patients used alcohol at the time of TB diagnosis, of whom 54% exhibited an AUD [21].

The co-occurring epidemics of TB and AUD in India, which contains a quarter of the world’s TB burden [1], provides an opportunity to study alcohol’s impact on TB disease in a high TB burden setting. We analyzed data from a clinically and demographically well-characterized cohort from the southern Indian states of Puducherry and Tamil Nadu to assess the association between alcohol use and clinical presentation at the time of TB diagnosis. We hypothesized that alcohol consumption, particularly at levels indicating risk for AUD, would be associated with worse clinical performances at the time of TB diagnosis.

## Methods

### Study population

Study participants were part of the Regional Prospective Observational Research for TB (RePORT) study, an ongoing prospective observational study in Puducherry and the Villupuram and Cuddalore districts of Tamil Nadu in southern India described in detail previously [22]. New acid fast bacilli (AFB) smear- and culture-positive pulmonary TB patients ≥ 6 years of age, receiving care through the Revised National TB Control Programme (RNTCP), who met the study’s eligibility criteria (S1 Table) were enrolled. Inclusion age criteria ≥ 6 years was based on the fact that isoniazid prophylaxis in India is recommended for exposed household contacts≤ 6 years of age, therefore they would not be eligible to partake in our research study evaluating TB risk. Exclusion criteria including multidrug resistant (MDR) TB cases at time of diagnoses, known household contact of MDR case, or previously treated for tuberculosis greater than one week in preceding thirty days, were excluded from the study. See S1 Table for further details.

### Data collection and study definitions

Study staff administered questionnaires on demographic and clinical characteristics, including age, gender, marital status, education level, and smoking status. Interviews were conducted at a clinic or the participant’s home. For all non-pregnant participants enrolled after 18 May 2016, a chest radiograph was conducted at baseline. While not required from RNTCP protocol, MGIT cultures were additionally obtained for all patients to ensure confirmation of tuberculosis diagnoses.

Alcohol use was assessed with the AUDIT-C (Alcohol Use Disorders Identification Test), a validated three question subset of AUDIT used to identify patients with hazardous drinking behavior or AUDs [23]. An AUDIT-C score of ≥four for men and ≥three for women was considered at-risk for an AUD (i.e., at-risk drinkers); the difference in scores per gender has been supported by previous studies and the National Institute for Alcohol Abuse and Alcoholism (NIAAA) for better detection for at-risk drinking [24–26]. Participants below these cut-offs, including those who abstained from drinking alcohol, were categorized as not at-risk. We also compared participants who reported drinking any alcohol to those who reported abstinence (i.e., non-drinkers). Participants were categorized as non-smokers, former smokers, or current tobacco smokers based on self-report. We stratified age into the following categories: 15–29, 30–44, 45–59, and ≥60 years. Diabetes was defined as random blood sugar >200 mg/dL or a self-reported diagnosis. Malnutrition was defined as having a body mass index (BMI) ˂18.5 kg/m^2^. We defined delay in accessing care as cough lasting ≥four weeks at study enrollment.

TB disease severity at the time of presentation, our outcome of interest, was assessed four ways: 1) Percent lung affected on chest radiograph, 2) presence of cavitation on chest radiograph, 3) AFB smear grade, and 4) time to positivity (TTP) on liquid mycobacterial growth indicator tubes (MGIT) culture. Two pulmonologists who had completed an NIH training on interpreting chest radiographs evaluated the extent of disease and the presence of cavitation using a standardized scoring form via Ralph et al [4]. For any discrepancies in the percent lung affected, both reviewers reviewed them together to decide on an adjudicated result. AFB smears of 1+ were categorized as low smear grade, while 2+ or 3+ smears were considered high grade. Baseline TTP, reflecting bacterial burden and infectiousness [27] was reported in days.

### Data management and analysis

**Fig 1:**
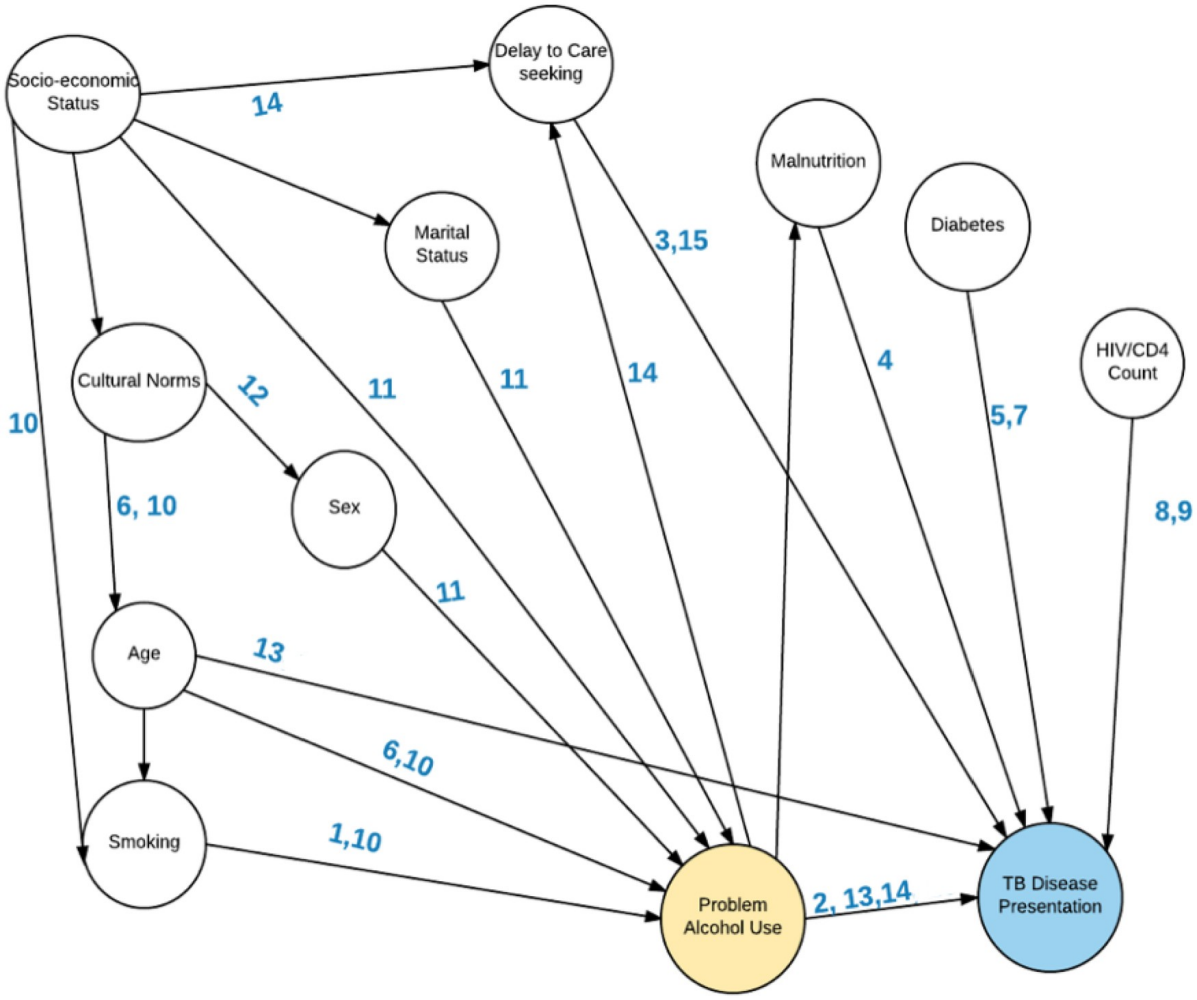
Directed Acyclic Graph (DAG) addressing factors related to alcohol use and tuberculosis disease presentation. Numbers on the arrows are citations in the reference list for this paper [41–49].

We assessed the association between potential socio-demographic risk factors and each aspect of disease severity through univariable analysis, stratified in two ways: by alcohol use (yes/no) and by AUD risk (at risk/not at risk). We assessed categorical variables using Fisher’s exact test, continuous normal variables with Student’s t-test (mean and standard deviations), and non-normal continuous variables with the Wilcoxon rank sum test (median and interquartile range). Odds ratios and corresponding 95% confidence intervals (CI) were calculated for categorical variables. Logistic regression was used to estimate the independent effect of alcohol use and AUD risk on smear grade and lung cavitation, controlling for known confounders of age and sex (Fig 1). Linear regression was used to estimate the independent effect of alcohol use and AUD risk on percentage of lung affected and TTP. Variables yielding p-values of ≤0.2 in univariable analysis were entered into model selection, including malnutrition (BMI <18.5), smoking history, educational status, diabetes, delayed access to care, and educational status. Variables found to change the effect estimate during backward elimination by >10% were considered confounders and were included in the final multivariable models. Interaction terms between age and delay in accessing care, as well as alcohol use and delay in accessing care, were tested in each multivariable model, but yielded no significant results. We analyzed data using SAS 9.4 (Cary, NC, USA).

### Ethical considerations

This study was approved by the Boston University Medical Campus Institutional Review Board and the Jawaharlal Institute of Postgraduate Medical Education and Research Ethics and Scientific Advisory Committees. Written informed consent was obtained from all study participants.

## Results

### Demographics

Data were analyzed for participants recruited from May 2014 through October 2017. Of 1344 patients screened, 1166 (86.8%) were enrolled (Table 1). Of 1166 enrolled, 691 reported drinking alcohol (59.3%) and 518 (75.0%) of those that reported drinking were at-risk drinkers. The mean age of the participants was 44.2 years (standard deviation [SD] 14.5) and 914 (78.4%) were male (Table 1). The largest proportions of participants who reported drinking alcohol or at-risk drinking were aged 45–59 years (45.0% and 47.1% respectively). All but one participant who reported consuming alcohol were male. Participants at-risk for AUD were more likely to be malnourished compared to other participants (70.2% vs. 54.2%, p<0.0001) (Table 1). Participants who reported any drinking were more likely to smoke than non-drinkers (37.6% vs. 5.3%, p <0.0001). The same association for current smokers was found between at-risk drinkers and not at-risk drinkers (43.4% vs. 9.3%, p<0.0001).

**Table 1:** Descriptive characteristics of new smear-positive pulmonary tuberculosis patients, stratified by any alcohol consumption and at-risk for alcohol use disorder (N = 1166), n (%).

Patient Characteristics	Drinkers (n = 691)	Non-Drinkers (n = 475)	P-value	At Risk Drinkers (n = 518)	Not at Risk (n = 648)	P-value
Sex						
Male	689 (99.7)	225 (47.4)		517 (99.8)	397 (61.3)	
Female	2 (0.3)	250 (52.6)	<0.0001	1 (0.2)	251 (38.7)	<0.0001
Age (years)						
15–29	44 (6.4)	168 (35.6)		18 (3.5)	194 (30.1)	
30–44	244 (35.3)	94 (19.9)		186 (35.9)	152 (23.6)	
45–59	311 (45.0)	123 (26.1)		244 (47.1)	190 (29.5)	
60+	92 (13.3)	87 (18.4)	<0.0001	70 (13.5)	109 (16.9)	<0.0001
Marital Status						
Never Married	61 (8.8)	138 (29.1)		34 (6.6)	165 (25.5)	
Married/Living Together	574 (83.1)	268 (56.4)		443 (85.5)	399 (61.6)	
Separated/ Divorced	31 (4.5)	15 (3.2)		25 (4.8)	21 (3.2)	
Widowed	25 (3.6)	50 (10.5)	<0.0001	16 (3.1)	59 (9.1)	<0.0001
Years of Education ǂ	6.2 (4.1)	8.3 (5.1)	<0.0001	5.8 (4.0)	8.0 (4.9)	<0.0001
BMI						
≥18.5 kg/m^2^	232 (33.9)	217 (45.8)		153 (29.8)	296 (45.8)	
<18.5 kg/m^2^	453 (66.1)	257 (54.2)	<0.0001	360 (70.2)	350 (54.2)	<0.0001
Diabetes Mellitus						
Yes	252 (36.5)	179 (37.7)		167 (32.2)	264 (40.7)	
No	439 (63.5)	296 (62.3)	0.67	351 (67.8)	384 (59.3)	0.0028
Tobacco Smoking Status						
Never smoker	180 (26.1)	418 (88.0)		114 (22.0)	484 (74.7)	
Former smoker	251 (36.3)	32 (6.7)		179 (34.6)	104 (16.0)	
Current smoker	260 (37.6)	25 (5.3)	<0.0001	225 (43.4)	60 (9.3)	<0.0001
Disease Characteristics						
Smear Status						
High	466 (67.9)	285 (60.2)		347 (67.5)	404 (62.6)	
Low	220 (32.1)	188 (39.8)	0.0072	167 (32.5)	241 (37.4)	0.084
Time to Positive MGIT (Days) ǂ	8.4 (4.0)	8.7 (4.0)	0.15	8.4 (4.0)	8.6 (4.0)	0.32
Delay in Care						
Yes	509 (73.7)	327 (68.8)		387 (74.7)	449 (69.3)	
No	182 (26.3)	148 (31.2)	0.073	131 (25.3)	199 (30.7)	0.041

^*ǂ*^ Mean and standard deviations reported for normal continuous variables from Student’s T test

BMI = Body mass index

MGIT = Mycobacterial growth indicator tube

### Disease severity characteristics by drinking status

Overall, 389 (33.3%) participants had chest radiographs available (S2 Table). The demographic and health information were generally comparable between those with and without chest radiographs, with a few differences. Comparing those participants with chest radiographs to those without, the former had a higher prevalence of diabetes (42.2% vs 34.4%, p = 0.0093), and more had high sputum smear grade (69.7% vs 62.3%; p = 0.014) (S2 Table).

Of the 389 with chest radiographs, 281 (79.4%) had cavitary disease and mean percent lung affected was 30.2% (SD 17.1%). A total of 138 (81.2%) at-risk drinkers had cavitary disease, compared to 143 (77.7%) not at-risk (p = 0.42). Mean percent lung affected was 33.5% (SD 17.7%) among at-risk drinkers compared to 27.2% (SD 16.1%) among participants not at-risk (p = 0.0005). Most (751/1166; 64.4%) participants had high smear grade; mean MGIT TTP was within a range of 8.4 to 8.7 days in all groups. Of at-risk drinkers, 347 (67.5%) had high smear grade compared to 404 (62.6%) not at-risk drinkers (p = 0.084, Table 1).

Table 2 shows the results of univariable and multivariable analyses assessing the association between alcohol use and clinical presentation. Any alcohol use was associated with an increased percent lung affected (difference = 8.9, p<0.0001). After adjusting for sex, age, malnutrition, smoking history, and educational status, the adjusted difference (10.8%) remained statistically significant (p<0.0001). The presence of cavitation did not differ between drinkers and non-drinkers in univariable analysis (OR = 0.9, 95% CI: 0.5–1.5) or after adjusting for sex, age, malnutrition, and diabetes in the multivariable analysis (aOR = 0.8, 95% CI: 0.4–1.8). People who drank any alcohol had increased odds of having a high smear grade compared to non-drinkers (OR = 0.7, 95% CI: 0.6–0.9, p = 0.0072). However, after adjusting for sex, age, diabetes, smoking, and delayed access to care, high smear grade did not differ between drinkers and non-drinkers (aOR = 1.0, 95% CI: 0.7–1.4, p = 0.98). TTP did not differ between drinkers and non-drinkers (difference = 8.5 hours, p = 0.15), which remained statistically non-significant after adjusting for age and sex (p = 0.51).

**Table 2:** Univariable & multivariable linear regression analyses on relationship between any alcohol use and various tuberculosis outcomes, reference group is non-drinkers.

Outcome	N	Univariate	P-value	Multivariate	P-value
Mean Difference in Percent Lung Affected, % (95% CI) *	354	8.9 (5.4, 12.4)	<0.0001	10.8 (8.3, 13.4)	<0.0001
Presence of Cavitation, OR (95% CI) **	354	0.9 (0.5, 1.5)	0.69	0.8 (0.4, 1.8)	0.63
High Smear Grade Status, OR (95% CI) ǂ	1156	0.7 (0.6, 0.9)	0.0072	1.0 (0.7, 1.4)	0.98
Mean Difference in *Hours* to Positivity for MGIT (95% CI) °	1101	8.5 (3.1, 20.1)	0.15	5.2 (-2.8, 13.1)	0.51

CI = confidence interval

OR = odds ratio

MGIT = Mycobacterial growth indicator tube

* Adjusted for sex, age, malnutrition (BMI <18.5), smoking history, and educational status

** Adjusted for sex, age, malnutrition, and diabetes

^*ǂ*^ Adjusted for sex, age, diabetes, smoking, and delayed access to care

° Adjusted for sex and age

### Disease severity characteristics by risk for AUD

People at-risk for AUD had significantly higher percentages of the lung affected than those not at-risk (difference = 6.3%, p value = 0.0005; Table 3). However, after adjusting for sex, age, malnutrition, smoking history, and educational status, the association became non-significant (3.7%, p value = 0.11). In adjusted analyses, the presence of cavitation did not differ between those at-risk and not at-risk for AUD after adjusting for sex, age, malnutrition, and diabetes (aOR = 1.1, 95% CI: 0.6–2.0). Outcomes such as high smear grade (OR = 0.8, 95% CI: 0.6–1.0) as well as TTP (difference = 5.8 hours, p = 0.32) did not show a statistically significant correlation with at-risk drinkers after adjusted analyses (Table 3).

**Table 3:** Univariable & multivariable linear regression analyses on relationship between being at risk for alcohol use disorder (AUD) and various outcomes, reference group is non-drinkers.

Outcome	N	Univariate	P-value	Multivariate	P-value
Mean Difference in Percent Lung Affected, % (95% CI) *	354	6.3 (2.8, 9.8)	0.0005	3.7 (1.4, 6.0)	0.11
Presence of Cavitation, OR (95% CI) **	354	0.8 (0.5, 1.4)	0.42	1.1 (0.6, 2.0)	0.88
High Smear Grade Status, OR (95% CI) ǂ	1156	0.8 (0.6, 1.0)	0.084	0.9 (0.6, 1.2)	0.38
Mean Difference in *Hours* to Positivity for MGIT (95% CI)	1101	5.8 (-5.7, 17.2)	0.32	0.7 (-6.1, 7.5)	0.92

CI = confidence interval

OR = odds ratio

MGIT = Mycobacterial growth indicator tube

* Adjusted for sex, age, malnutrition (BMI <18.5), smoking history, and educational status

** Adjusted for sex, age, malnutrition, and diabetes

^*ǂ*^ Adjusted for sex, age, diabetes, smoking, and delayed access to care

° Adjusted for sex and age

## Discussion

Our study of smear-positive pulmonary TB patients in two southern Indian states found a very high prevalence of AUD risk among our cohort, with more than 44% of participants classified as at-risk, compared to 9% nationally in India [28]. Alcohol use was associated with an increase in the proportion of lung affected at the time of TB diagnosis, which may have implications for short-term outcomes and chronic sequelae of TB. Neither alcohol use nor the risk of AUD was associated with cavitation, sputum smear grade, or bacterial burden (as assessed by TTP).

Previous *in vivo* and in *vitro* studies have suggested that alcohol use significantly disrupts the immune response, increasing susceptibility to respiratory diseases such as tuberculosis [29–30]. Alcohol abuse impairs phagocytic function of monocytes and alveolar macrophages, which serve an essential function in destroying the majority of inhaled pathogens [30, 31]. Such initial defense is critical in clearing and preventing mycobacteria from further proliferation. For chronic alcohol users, while the number of monocytes eventually increase as compensation, their functioning remains impaired–whether through decreased adherence to other cells, production of reactive oxygen species, or alterations in various protein expression on monocyte surfaces [29].

Researchers previously hypothesized that heavy alcohol use would be associated with infectiousness among TB patients due to increased cavitation and a higher likelihood of smear-positivity [10, 32]. Previous studies found an approximately 25% increased risk of cavitation among those who use alcohol and a similar increased risk of smear positivity [9–10] However, these studies were done in low-burden settings and only one assessed alcohol use with a validated instrument. The only prior study which used standardized questionnaires for frequency and quantity of alcohol consumption found no independent relationship between recent alcohol use or lifetime alcohol use and lung cavitation at the time of TB diagnosis [33]. Some studies have found alcohol use to be associated with transmission and case clustering [10], supporting alcohol’s potential association with infectiousness. However, this finding has been inconsistent across studies [2]. Whether this is a behavioral mechanism, with patients who consume heavy amounts of alcohol or exhibit risky alcohol behavior being more likely to delay seeking care when they become ill [18], or a biologic mechanism, where the immune effects of alcohol may lead to an accelerated course and more aggressive presentation of the disease, remains poorly understood.

The relationship between alcohol use and disease severity is complicated by additional comorbidities and demographics that are associated with TB disease. For instance, while diabetes has been shown to be associated with cavitation and smear positivity, HIV often has the opposite effect [34]. Whether alcohol may have an antagonistic, additive, or synergistic effect with these comorbidities or with other demographics is unclear. The four previous studies [9–11, 33] that investigated alcohol and TB disease severity were in populations where women consume alcohol; one study reported 15% of those who consumed alcohol were female [10] compared to <1% in our cohort. Similarly, only one study reported comorbid diabetes [33]; 19% of their cohort had diabetes compared to 34% of our cohort. In that study, among patients who drank alcohol one month as well as six months preceding their TB diagnoses, comorbid diabetes was consistently found to be associated with chest cavitation [33].

A strength of our analysis was controlling for delayed access to care and known confounders of the association between alcohol and disease severity (Fig 1), but unmeasured confounding is possible. Furthermore, our use of the validated AUDIT-C instrument addressed the limitation of many previous studies that used varied and poor definitions of alcohol use. In the future, other instruments, such as the full AUDIT or Alcohol Timeline Followback, could add more granularity to studies of alcohol use and TB disease [35–37].

Our study has limitations. First, all study participants were sputum smear-positive at diagnosis, so we were unable to assess a relationship between alcohol consumption and any smear positivity. However, we do show that alcohol use was not associated with a higher smear grade. Another limitation is that smear positivity has also been shown to be associated with lung cavitation [38], which may have limited our ability to see any additional effect that alcohol use, particularly at heavy levels, may have on cavitation. Additionally, cavitation has been associated with both higher bacterial burden (i.e., shorter TTP) among smear-positive pulmonary TB patients [39] and with higher smear grade [40]. Stratifying alcohol consumption by quality of alcohol and origin of production were not accounted for in this study; further studies may consider delineating these characteristics. Chest radiograph interpretation is also difficult, both in determining the extent of disease and the presence of cavitation, thus serving as a limitation in this study. However, we attempted to address this limitation by requiring consensus between two trained readers and following standard protocols [4]. Using a chest CT may be more sensitive in detecting cavities than chest radiograph, and may be helpful for future studies.

Our study highlights an association between the proportion of the lung affected by TB disease and alcohol consumption, particularly when comparing individuals who report any drinking with abstainers. We found high levels of alcohol use and AUD risk among our male pulmonary TB patients. That alcohol use appears to modify disease presentation warrants further evaluation and supports the need to address alcohol use in patients with TB. Further studies may consider delineating these effects and should consider the hepatotoxic effects of concurrent alcohol and anti-TB medications.

## Supporting information

S1 Table Eligibility criteria for RePORT cohort index cases. (DOCX)

S2 Table Descriptive characteristics of cases with and without chest radiographs (N = 1169). (DOCX)

S1 File (ZIP)
