# Supplementary material for: Alcohol use and tuberculosis clinical presentation at the time of diagnosis in Puducherry and Tamil Nadu, India
Source: PLoS One. 2020 Dec 17;15(12):e0240595. doi: 10.1371/journal.pone.0240595 (PMC7746146; doi:10.1371/journal.pone.0240595)
Supplement: S1 Table — (DOCX) [file pone.0240595.s001.docx]

S1 Table. Eligibility criteria for RePORT cohort index cases

| Inclusion criteria | Exclusion criteria |
| --- | --- |
| - 6 years of age or greater - Positive sputum AFB smear result (≥1+ smear grade reading) - Culture positive for Mycobacterium tuberculosis (Mtb) - No history of TB treatment - Intended to complete TB therapy for the recommended duration - Planned to enroll in the DOTS program for treatment - Intended to reside in the study area for the duration of their treatment | - Refused an HIV test or did not have documentation of a HIV test within the past three months - On treatment for active TB for greater than one week in the preceding 30 days - Known to have multidrug-resistant TB (MDR-TB) or extensively drug-resistant TB (XDR-TB) at diagnosis - A known household contact of an MDR or XDR-TB case - Chose not to initiate or complete the treatment course - Received more than seven days of fluoroquinolone therapy or other drugs with anti-TB activity (e.g., rifampin, ethambutol, clofazimine, aminoglycosides) for any reason in the preceding 30 days - Were too sick to enroll |
