# Supplementary material for: Alcohol use and tuberculosis clinical presentation at the time of diagnosis in Puducherry and Tamil Nadu, India
Source: PLoS One. 2020 Dec 17;15(12):e0240595. doi: 10.1371/journal.pone.0240595 (PMC7746146; doi:10.1371/journal.pone.0240595)
Supplement: S2 Table — (DOCX) [file pone.0240595.s002.docx]

S2 Table: Descriptive characteristics of cases with and without chest radiographs (N=1169)

| ***Patient Characteristics*** | No Chest Radiograph n=777 | Chest Radiograph n=389 | P-value |
| --- | --- | --- | --- |
| Sex |  |  |  |
| Male | 606 (78.0) | 308 (79.2) |  |
| Female | 171 (22.0) | 81 (20.8) | 0.64 |
| Age (years) |  |  |  |
| 15-29 | 145 (18.7) | 67 (17.2) |  |
| 30-44 | 205 (26.5) | 133 (34.2) |  |
| 45-59 | 284 (36.7) | 150 (38.6) |  |
| 60+ | 140 (18.1) | 39 (10.0) | 0.0008 |
| BMI |  |  |  |
| ≥18.5 kg/m^2^ | 285 (37.0) | 164 (42.2) |  |
| <18.5 kg/m^2^ | 485 (63.0) | 225 (57.8) | 0.089 |
| Diabetes mellitus |  |  |  |
| Yes | 267 (34.4) | 164 (42.2) |  |
| No | 510 (65.6) | 225 (57.8) | 0.0093 |
| ***Disease Characteristics*** |  |  |  |
| Percent Lung Affected *ǂ* | -- | 30.2 (17.1) | *N/A* |
| Cavitation |  |  |  |
| Yes | -- | 281 (79.4) |  |
| No | -- | 73 (20.6) | *N/A* |
| Smear Status |  |  |  |
| High | 482 (62.3) | 269 (69.7) |  |
| Low | 291 (37.7) | 117 (30.3) | 0.014 |
| Time to Positivity (Days)*ǂ* | 8.5 (4.1) | 8.5 (3.9) | 0.85 |
| Delay in Accessing Care |  |  |  |
| Yes | 548 (70.5) | 288 (74.0) |  |
| No | 229 (29.5) | 101 (26.0) | 0.21 |
| ***Socio-Demographics*** |  |  |  |
| Marital Status |  |  |  |
| Never Married | 128 (16.5) | 71 (18.3) |  |
| Married/Living Together | 556 (71.6) | 286 (73.5) |  |
| Separated/Divorced | 28 (3.6) | 18 (4.6) |  |
| Widowed | 61 (7.9) | 14 (3.6) | 0.032 |
| Years of Education *ǂ* | 6.7 (4.7) | 7.7 (4.4) | 0.0003 |
| Number of Household Members *ǂ* | 4.0 (1.8) | 3.6 (1.4) | 0.47 |
| Alcohol User |  |  |  |
| Yes | 458 (58.9) | 233 (59.9) |  |
| No | 319 (41.1) | 156 (40.1) | 0.76 |
| At Risk Alcohol Users |  |  |  |
| Yes | 332 (42.7) | 186 (47.8) |  |
| No | 445 (57.3) | 203 (52.2) | 0.10 |
| Tobacco Smoking Status |  |  |  |
| Never smoker | 394 (50.7) | 204 (52.4) |  |
| Former smoker | 217 (27.9) | 66 (17.0) |  |
| Current smoker | 166 (21.4) | 119 (30.6) | <0.0001 |
| Municipality |  |  |  |
| Cuddalore | 243 (31.3) | 35 (9.0) |  |
| Puducherry | 366 (47.2) | 305 (78.4) |  |
| Villupuram | 167 (21.5) | 49 (12.6) | <0.0001 |

*ǂ* Mean and standard deviations reported for normal continuous variables from Student’s T test
